# The Ecology of Economic Distress and Life Expectancy

**DOI:** 10.3389/ijph.2024.1607295

**Published:** 2024-07-26

**Authors:** William Brinson Weeks, Ji E. Chang, José A. Pagán, Elizabeth Adamson, James Weinstein, Juan M. Lavista Ferres

**Affiliations:** ^1^ Microsoft, AI for Good Lab, Redmond, WA, United States; ^2^ School of Global Public Health, New York University, New York, NY, United States; ^3^ Novartis Foundation, Basel, Switzerland; ^4^ Microsoft Research, Redmond, WA, United States

**Keywords:** social determinansts of health, life expectancy, local economic prosperity, rurality, racial disparities

## Abstract

**Objectives:**

To determine whether life expectancy (LE) changes between 2000 and 2019 were associated with race, rural status, local economic prosperity, and changes in local economic prosperity, at the county level.

**Methods:**

Between 12/1/22 and 2/28/23, we conducted a retrospective analysis of 2000 and 2019 data from 3,123 United States counties. For Total, White, and Black populations, we compared LE changes for counties across the rural-urban continuum, the local economic prosperity continuum, and for counties in which local economic prosperity dramatically improved or declined.

**Results:**

In both years, overall, across the rural-urban continuum, and for all studied populations, LE decreased with each progression from the most to least prosperous quintile (all *p* < 0.001); improving county prosperity between 2000–2019 was associated with greater LE gains (*p* < 0.001 for all).

**Conclusion:**

At the county level, race, rurality, and local economic distress were all associated with LE; improvements in local economic conditions were associated with accelerated LE. Policymakers should appreciate the health externalities of investing in areas experiencing poor economic prosperity if their goal is to improve population health.

## Introduction

In the United States (US), rural-urban disparities in population characteristics, health status, and health outcomes have been long established, with rural-dwelling individuals more likely to be White, older, and poorer, and to have riskier health behaviors, worse health status, higher rates of potentially avoidable hospitalizations, and higher mortality rates than urban-dwelling ones [1, 2]. The gap in life expectancy (LE) between rural and urban residents in the US is established and has been growing, with most of the growth in disparities being attributable to increasing cardiovascular disease prevalence in rural dwellers [3] and socioeconomic disparities between rural- and urban-dwellers [4], a disparity that is particularly evident among Medicaid enrollees [5].

The racial makeup of the local population has also been implicated as an explanation of rural-urban disparities in LE, with lower LE at age 25 among non-White rural dwellers [6]. Further, rural-dwelling racial/ethnic minorities are more likely than White counterparts to have poorer health status, to be obese, and to be unable to see a physician in the last 12 months because of costs [3].

There is also longstanding evidence that socioeconomic status is associated with health and health outcomes at the individual level [7, 8]. Recent studies have found that local economic prosperity at the geographic level is also associated with health status, health outcomes, and healthcare quality [9, 10]. Importantly for policymakers, improvements in geographically defined local economic conditions are associated with improved cardiovascular outcomes among 45–64 year olds [11] and mortality rates among older Medicare enrollees [12]. It is unclear how LE disparities across race categories, the rural-urban continuum, and local economic prosperity have evolved over time as a result of changes in local economic prosperity.

This study assesses the relationship between rurality, race, cross-sectional local economic prosperity, changes in local economic prosperity, cross-sectional LE, and changes in LE over time. Understanding these relationships in tandem are critical, especially given rising levels of income inequality in the US, particularly in rural areas alongside racial/ethnic disparities in health outcomes. Our approach prioritizes the perspective that the lived experiences of those communities over attempts to adjust for the drivers of those lived experiences. We used county-level data to compare information from 2000 to 2019 on LE and changes in LE and, thus, better understand how economic prosperity may be related to population health.

## Methods

### Data

We obtained county-specific LE at birth estimates for the entire county population, the White county population, and the Black county population for 2000 and 2019 from the Institute for Health Metrics and Evaluation [13]. We used the 2013 Urban-Rural Classification Scheme for Counties (based on the 2010 Census) [14] from the Centers for Disease Control and Prevention’s National Center for Health Statistics to classify counties as metro (codes 1 and 2), suburban (codes 3 and 4), or rural (codes 5 and 6) and linked those codes to LE statistics using county Federal Information Processing Standards (FIPS) codes. We also obtained county-level distressed community index (DCI) scores, population estimates, and percentages of the population that is Black or White for 2000 and 2019 from the Economic Innovation Group (EIG) [15]. Constructed from seven measures of local economic distress (including the prime-age unemployment rate, the change in number of jobs and employers in the past 5 years, the proportion of adults without a high school diploma, the housing vacancy rate, the poverty rate, and the median income ratio (the median household income as a share of the metro area (or state, for non-metro areas) median household income) collected from the US Census, US Bureau of Labor Statistics, and the American Community Survey, DCI scores are ranked percentiles that are equally distributed, range from 0 (most prosperous) to 100 (least prosperous), and are frequently compared as quintiles of equivalent numbers of counties [15].

Linking county-level LE estimates, urban-rural classification, and EIG data for both years resulted in data for 3,123 counties in the 50 United States and Washington, DC, representing an estimated 325,610,413 people in 2019. Because data were publicly available and aggregated at the county level, human subjects review was not required.

### Analysis

We first examined cross-sectional 2000 and 2019 LE for the entire (including White, Black, and other), White, and Black populations living in metro, suburban, and rural counties, across prosperity quintiles. We subtracted 2000 from 2019 DCI scores to classify counties into quintiles that experienced a major improvement in local economic conditions (experiencing a mean DCI decrease of 22.0 points), a minor improvement in local economic conditions (a mean DCI decrease of 6.2 points), no change in local economic conditions (a mean DCI increase of 0.5 points), a minor decline in local economic conditions (a mean DCI increase of 7.1 points), or a major improvement in local economic conditions (a mean DCI increase of 21.0 points) in relative local economic prosperity between 2000 and 2019 (Table 1). We compared changes in LE for each population living in each setting according to change in prosperity.

**TABLE 1:** Distribution of numbers of counties, change in distressed community index scores, and 2019 population size estimates according to change in distressed community index scores between 2000 and 2019 (United States, 2000 and 2019).

Category of relative change in local economic prosperity	Number of counties	Change in distressed community index score		2019 population estimates		
		Mean	Range	Entire	White	Black
Major improvement	625	(22.0)	(71) – (11)	47,609,981	24,358,138	7,560,950
Minor improvement	625	(6.2)	(11) – (2)	88,183,894	48,634,983	11,115,678
No change	623	0.5	(2) – 3	104,116,969	65,368,752	9,954,455
Minor decline	625	7.1	3–11	53,179,844	35,935,107	6,976,150
Major decline	625	21.0	11–53	32,519,738	23,410,892	4,439,498

Because we were interested in differences in populations and not counties, we weighted year-, county-, and race-specific LE by year-, county- and race-specific population estimates and, as with previous analyses [9, 10, 12, 16], made comparisons using Analysis of Variance (ANOVA). We used SPSS v 28 (released 2022, Armonk, NY: IBM Corporation) for all analyses.

## Results

**FIGURE 1:**
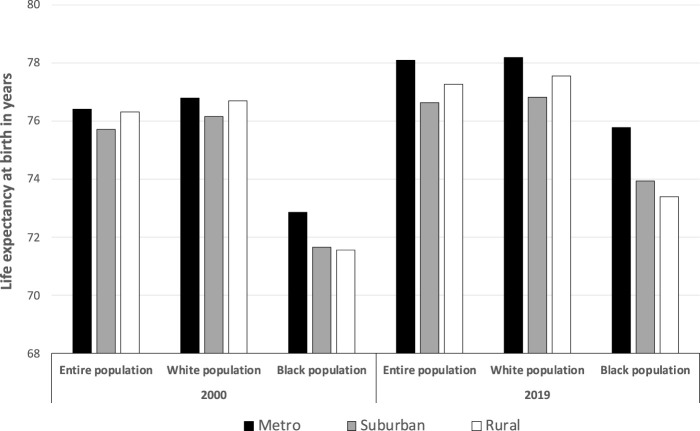
Life expectancy at birth, by race and rural status, 2000 and 2019 (United States, 2000 and 2019).

While metro county residents invariably had longer LEs than residents of rural or suburban counties across all three population groups (*p* < 0.001 for all), differences were more dramatic in 2019 than in 2000 (Figure 1). Across all rural-urban classifications, Black residents experienced a greater increase in LE between 2000 and 2019 (1.84, 2.28, and 2.93 years for rural, suburban, and metro county dwellers, respectively) than White residents (0.85, 0.68, and 1.39) or the entire population (0.94, 0.93, and 1.70). Only among the Black population was there a clear longevity advantage when moving to increasingly urban areas.

**FIGURE 2:**
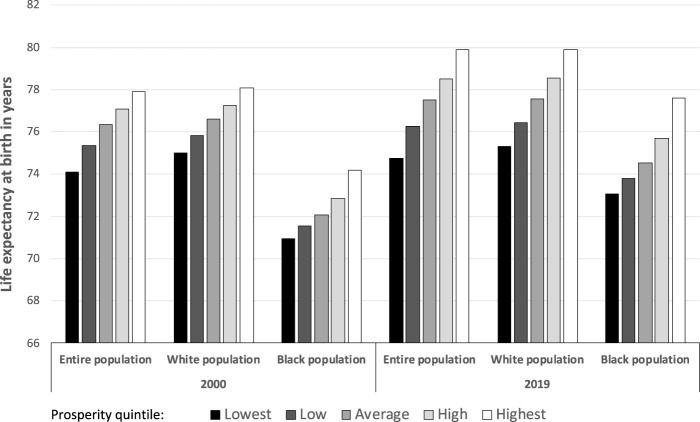
Life expectancy at birth, by race and local economic prosperity quintile, 2000 and 2019 (United States, 2000 and 2019).

When comparing LE across prosperity quintiles, we found increased longevity as prosperity increased (all *p* < 0.001) (Figure 2). Between 2000 and 2019, LE differences between lowest and highest prosperity quintile increased for all racial groups examined (from 3.79 to 5.16 years for the entire population; from 3.09 to 4.61 for White populations; and from 3.26 to 4.54 for Black populations). For each prosperity quintile, the entire population and White residents invariably had higher LEs than Black residents.

**FIGURE 3:**
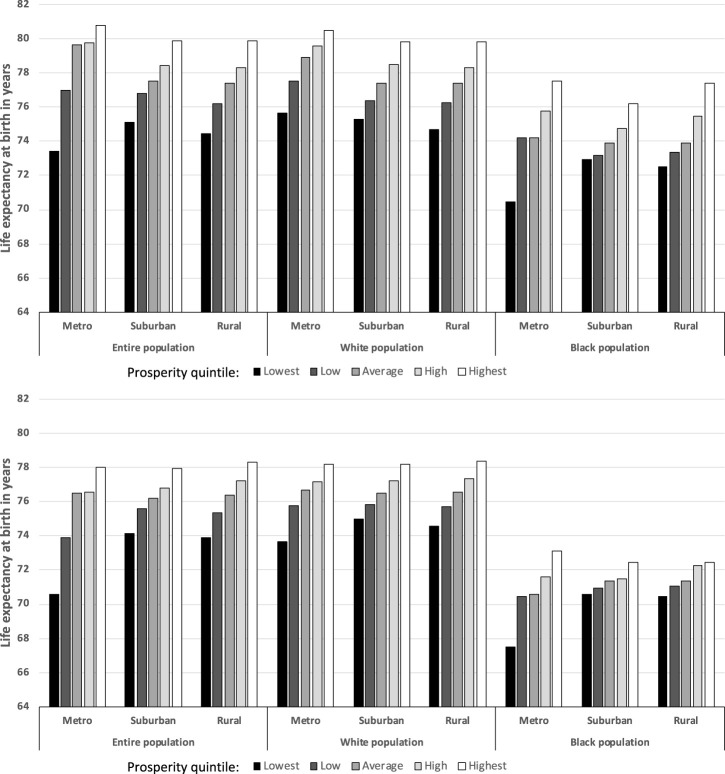
Life expectancy at birth in 2019 (top) and 2000 (bottom), for the entire, White, and Black populations by quintile of local economic prosperity (United States, 2000 and 2019).

When examining LE disparities across racial and economic prosperity groups within rural-urban classifications, with one exception (the 2019 difference between low and average prosperity among metro-dwelling Black populations), we found the same increase in longevity with increasing prosperity (all others, *p* < 0.001) (Figure 3). In 2019, disparities between the lowest and highest prosperity quintile were greatest for the entire urban population (7.39 years) and urban-dwelling Black populations (7.11 years) and were smallest for suburban- (3.27 years) and rural- (4.56 years) dwelling Black residents. With regard to changes in disparities in LE, between 2000 and 2019 across prosperity quintiles, rural-dwelling Black populations had a 2.96-year LE difference increase between the lowest and highest prosperity quintile, urban-dwelling Black populations had a 1.48-year increase, and suburban-dwelling Black populations had a 1.40-year increase. Between 2000 and 2019, the entire urban-dwelling population was the only demographic grouping that experienced a decrease (0.04 years) in disparities.

**FIGURE 4:**
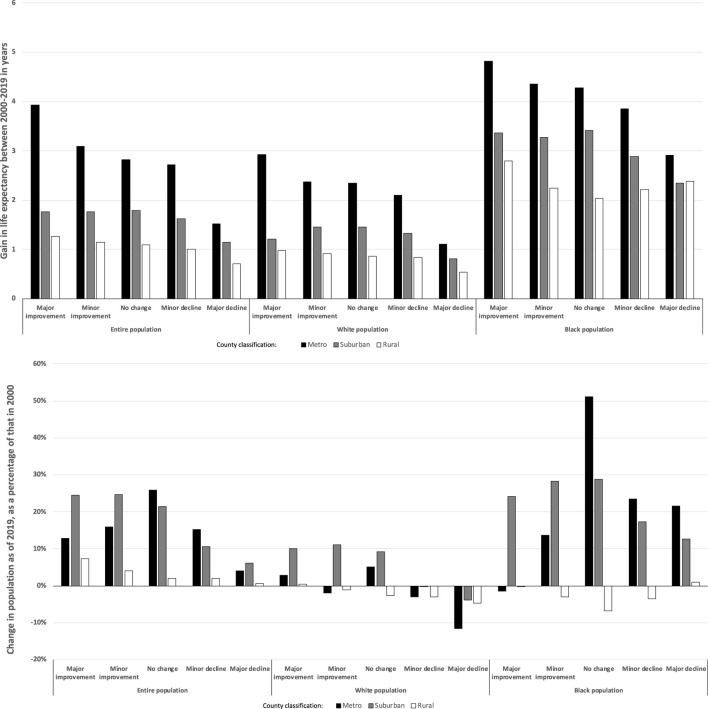
Change in life expectancy (top) and population size (as a percentage of 2000 population, bottom) between 2000 and 2019 by quintile of change in economic prosperity, race, and rural status (United States, 2000 and 2019).

When comparing race- and county-rural-classification-specific gains in LE between 2000 and 2019 as a function of whether the county experienced a relative major or minor improvement or decline in local economic prosperity between 2000 and 2019, we found that metro areas with major improvements in economic prosperity experienced the greatest gains in LE, with increasing lower gains when moving to major declines in local economic prosperity, for all racial categories (Figure 4, top). For the entire population and White residents, a similar, albeit less pronounced, pattern of decreasing LE gains was seen. While that pattern held for the entire population, there was no evident pattern for White or Black residents, though in both cases, LE gains were higher for those living in counties experiencing major improvements in prosperity than in those experiencing major declines in prosperity.

When examining the entire and White populations living in counties experiencing changes in economic prosperity, we found a relative increase of the size of the entire population in counties with improving or stagnant local economic conditions; for white populations, this was accompanied by a reduction in populations living in counties experiencing economic declines (Figure 4, bottom). These patterns were not seen in the Black population, where we found population growth – relative to overall population growth – in metro and suburban counties experiencing no economic change or declining economic conditions. Finally, when comparing the White to the Black populations, between 2000 and 2019, Black populations were more likely to experience a major (18.9% vs. 12.3%) improvement in local economic distress (Table 1).

## Discussion

For the entire, White, and Black populations, we found that metro county residents had longer LEs that rural or suburban county residents and that differences across the rural-urban continuum increased between 2000 and 2019. These findings are consistent with recent reports documenting a persistent and widening rural-urban gap in LE [3]. Further, we found persistent decreases in LE with decreasing county prosperity for all populations examined, with disparities between the least and most prosperous counties increasing between 2000 and 2019. Generally, the LE differences attributable to local economic prosperity that were evident overall persisted when comparing racial groups within rural-urban classifications. We found decreasing LE gains when comparing counties that experienced major improvement to those experiencing major declines in economic prosperity between 2000 and 2019. Finally, we found evidence of selective migration: the increasing size of the White populations that experienced improving local economic conditions was not seen in Black populations. While Black populations were more likely to experience a major improvement in local economic distress, the Black population living in areas with minor or major declines in economic prosperity grew substantially between 2000 and 2019.

These findings support the literature that increasingly attributes population health status to social determinants of health. Local economic conditions can impact health through various mechanisms [17]. Prior research identified widespread disparities in access to medical care in low-income and underserved communities [18]. Furthermore, residents of low-income areas are more likely to dwell in unhealthy or unsanitary housing conditions and exposed to air pollution [19]. Low-income neighborhoods also have disproportionately high crime rates, fewer green spaces, and less access to healthy foods [20]. These factors may work together to break down social cohesion and increase stress for low-income individuals and contribute to poorer health outcomes. Socio-economic and racial inequities persist in the U.S. due in part to differences in neighborhood environments. Non-Hispanic Black populations are not only more likely to experience poverty, they are also more likely to live in areas of concentrated poverty, and less likely to experience upward social mobility [21, 22]. Importantly, our findings suggest that local improvement in economic conditions is associated with relative gains in LE, with the urban Black population having the greatest incremental gains and rural dwellers experiencing the smallest incremental grains across racial and rural-urban categories.

To be sure, access to and improvements in healthcare may facilitate some of the longevity increases we saw. Because, as a measure, LE at birth is sensitive to deaths of infants, reduction in neonatal mortality in the recent past [23] may have driven the overall increases in LE that we found. However, those gains may have been partially offset by increases in maternal mortality during the study’s time period [24], which were worse (in San Antonio, Texas) for women living in low income ZIP Codes [25].

Addressing socioeconomic health disparities is a major US policy goal. While economic improvement has been linked with improvement in health status [11, 12], this study provides additional insights to health systems, health insurers, private companies, and policymakers who might want to understand potential population health and equity benefits of investing in areas experiencing poor economic prosperity for the purposes of improving population health. More studies are needed to further explore interactions and causal mechanisms underlying the relationship between local economic conditions and the health of populations.

Our study has several limitations. First, we used publicly available estimates of LE, definitions of rurality, and calculations of local economic distress; to the degree these estimates are inaccurate, our results will be, too. Second, we focused on LE at birth. While evaluation of LE at different ages might generate different results, those data were less available for all counties, particularly for the Black population. Third, we conducted our analysis at the county level. Analyses at more granular levels might generate different results [26]. Fourth, we were not able to include 36 sparsely populated counties in our analysis. Fifth, our analysis was largely qualitative in nature. While we performed some standard ANOVA testing, our intent was to document relative changes at the community level over the period examined – the lived experiences of those communities – with the sense that focusing on multiple tests of statistical significance, adjustment for the very factors that we are examining, or modifying results by social determinants that likely account for our findings would diminish those lived experiences. Sixth, we used a simple method for comparing change in local economic distress over time – calculation of changes in the DCI scores. DCI scores capture relative economic distress; however, in a time of economic change, relative, as opposed to absolute, changes in local economic distress may be more relevant, particularly when considering physical and mental health [27]. Seventh, we categorized those relative changes into quintiles of improvement or decline for analytic purposes; different classification schemes could generate different results. Finally, our findings are associative and not causative.

Our findings have implications for those who would like to leverage changes in social determinants of health in order to improve population health. While focusing on particular social determinants may be effective at improving population health [28], encouraging economic growth and prosperity in targeted areas may also be a very effective way of improving population health without being prescriptive on specific policies targeting social determinants of health.
